# Treatment outcomes for cluneal neuropathy: a systematic review and meta-analysis

**DOI:** 10.1016/j.bas.2026.106062

**Published:** 2026-04-27

**Authors:** Kristina Catalano, Lukas Andereggen, Gerrit A. Schubert, Markus Bruder, Julien N. Jost

**Affiliations:** a Department of Neurosurgery, Cantonal Hospital Aarau, Aarau, Switzerland; b Faculty of Medicine, University of Bern, Bern, Switzerland; c Department of Neurosurgery, RWTH Aachen University, Aachen, Germany; d Department of Neurosurgery, Goethe University Frankfurt am Main, Frankfurt, Germany

**Keywords:** Cluneal nerves, Entrapment neuropathy, Surgery, Injection therapy, Radiofrequency ablation, Peripheral nerve stimulation

## Abstract

**Background:**

Cluneal nerve entrapment is an increasingly recognized but often overlooked cause of chronic low back pain. Various treatment strategies have been described, including surgical decompression, injection therapy, radiofrequency ablation (RFA), and peripheral nerve stimulation (PNS). However, pooled evidence on treatment outcomes remains limited.

**Objective:**

This systematic review and meta-analysis aimed to evaluate pooled responder rates following different treatment modalities for cluneal nerve-related pain.

**Methods:**

A systematic review and meta-analysis was performed of studies reporting dichotomous responder outcomes after treatment of superior or middle cluneal nerve entrapment. Responder status was defined as ≥50% pain reduction or study-defined clinical improvement. Random-effects meta-analysis of proportions with logit transformation was used to pool responder rates. The primary analysis included treatment arms with follow-up of ≥3 months, and a predefined sensitivity analysis was restricted to studies with follow-up of ≥6 months. Subgroup analyses were performed by treatment modality and by surgical versus non-surgical treatment strategy.

**Results:**

A total of 26 treatment arms including 628 patients were analyzed. The pooled responder rate was 78.3% (95% CI 72.9-82.9) at ≥3 months and 82.9% (95% CI 75.3-88.6) at ≥6 months. Stratified by treatment modality, pooled responder rates were 93.1% (95% CI 84.9-97.0) for surgical decompression, 72.4% (95% CI 65.9-78.1) for injection therapy, 75.1% (95% CI 62.1-84.7) for radiofrequency ablation, and 80.1% (95% CI 58.5-92.0) for peripheral nerve stimulation.

**Conclusion:**

Targeted treatment of cluneal nerve entrapment was associated with high pooled responder rates at both ≥3 and ≥ 6 months of follow-up. Surgical decompression was associated with the highest pooled responder proportion; however, differences between treatment modalities should be interpreted cautiously given the predominantly observational, non-comparative nature of the available evidence, heterogeneity in diagnostic criteria and outcome definitions, and the potential for confounding by indication. Prospective comparative studies using standardized diagnostic and outcome measures are needed.

## Highlights

• Cluneal neuropathy is an under-recognized cause of chronic low back pain.
• Pooled responder rates across treatments are high in observational studies.
• Surgical cohorts show high response but reflect selected patient populations.
• Diagnostic pathways and use of nerve blocks vary substantially across studies.
• Findings represent observational evidence rather than comparative effectiveness.
• Standardized diagnostic criteria and reporting are needed for future studies.

## 1 Introduction

Chronic low back pain (LBP) represents one of the leading causes of disability worldwide and poses a substantial socioeconomic burden. While degenerative spinal disorders account for a large proportion of cases, a considerable number of patients suffer from extra-spinal pain generators that may remain underdiagnosed. Among these, cluneal nerve entrapment (CNE) has increasingly been recognized as a potentially treatable cause of chronic LBP. (Fujihara et al., 2024)

The cluneal nerves originate from the dorsal rami of the lumbar spinal nerves and provide sensory innervation to the posterior iliac crest and gluteal region. Two clinically relevant syndromes have been described: superior cluneal nerve (SCN) entrapment, typically occurring at the osteofibrous tunnel formed by the thoracolumbar fascia over the iliac crest, and middle cluneal nerve (MCN) entrapment, often related to compression near the long posterior sacroiliac ligament. These entrapment syndromes may produce localized LBP that can radiate to the buttock or posterior iliac crest and may clinically mimic more common spinal pathologies. (Wu et al., 2023; Bodner et al., 2016).

Although historically considered rare, increasing clinical awareness suggests that CNE may account for a meaningful proportion of patients with chronic LBP. Previous studies have estimated that SCN entrapment may be present in approximately 1-14% of patients with LBP, with some reports suggesting even higher prevalence among individuals with refractory symptoms. (Yamauchi et al., 2018; Takada et al., 2024).

A variety of treatment options have been described for CNE, ranging from minimally invasive interventional approaches, such as local anesthetic or steroid injections, radiofrequency ablation (RFA), and peripheral nerve stimulation (PNS), to surgical decompression of the entrapped nerve. (Wu et al., 2023; Bodner et al., 2016; Wu et al., 2022; Luo and Zhao, 2024; Yildirim, 2024; Visnjevac et al., 2022; Lindley and Anders, 2024; Abd-Elsayed and Gyorfi, 2024; Mas et al., 2025; Skaribas et al., 2025)While individual case series and cohort studies have reported favorable outcomes with these treatments, the overall effectiveness across modalities remains uncertain. Furthermore, the durability of treatment response beyond short-term follow-up has not been systematically evaluated.

Given the increasing recognition of CNE as a treatable source of chronic LBP, a quantitative synthesis of treatment outcomes is warranted. (Isu et al., 2018; Kim et al., 2022a, 2024) Therefore, the aim of this systematic review and meta-analysis was to estimate pooled responder rates after surgical and interventional treatment of SCN and MCN entrapment, and to evaluate the durability of treatment response at follow-up thresholds of ≥3 months and ≥6 months.

Across the included literature, diagnostic and therapeutic pathways were not uniform. In most clinical series, patients were initially identified based on characteristic clinical findings such as focal tenderness or trigger points along the posterior iliac crest or sacroiliac region, often followed by targeted diagnostic nerve blocks to increase diagnostic confidence. Less invasive treatments - including repeated injections, radiofrequency procedures, or peripheral nerve stimulation - were typically applied prior to considering surgical decompression, although the sequence and thresholds for escalation varied substantially between studies. This variability is clinically relevant because it introduces selection effects, with more invasive treatments often performed in patients with persistent symptoms and higher diagnostic certainty for cluneal nerve-mediated pain.

## 2 Material and methods

### 2.1 Study design

This systematic review and meta-analysis was conducted in accordance with the Preferred Reporting Items for Systematic Reviews and Meta-Analyses (PRISMA) guidelines. The review protocol was prepared a priori and submitted to the International Prospective Register of Systematic Reviews (PROSPERO); registration was pending at the time of manuscript submission. The review aimed to evaluate clinical outcomes of surgical and non-surgical treatments for superior and middle cluneal nerve entrapment.

### 2.2 Literature search

A systematic literature search was conducted in PubMed/MEDLINE and the Cochrane Central Register of Controlled Trials (CENTRAL). The search strategy combined controlled vocabulary and free-text terms related to cluneal nerves and entrapment neuropathy, together with intervention-related terms including decompression, surgery, injection therapy, radiofrequency ablation, and peripheral nerve stimulation.

The full search strategy for each database is provided in Supplementary Material S2. In addition, the reference lists of included studies and relevant reviews were manually screened to identify additional eligible publications. The final search date is specified in the Supplement.

### 2.3 Eligibility criteria

Studies were eligible if they met the following criteria:

(1) involved patients with clinically suspected or confirmed superior or middle cluneal nerve entrapment;
(2) evaluated a therapeutic intervention targeting the cluneal nerves (e.g., surgical decompression, injection therapy, radiofrequency ablation, or peripheral nerve stimulation);
(3) reported clinical outcomes including pain reduction or clinically meaningful improvement; and
(4) provided sufficient data to determine the number of responders and total treated patients.

Case reports, narrative reviews, editorials, and purely anatomical studies without clinical outcome data were excluded. Studies not published in English or without extractable outcome data were also excluded.

### 2.4 Study selection

**Fig. 1:**
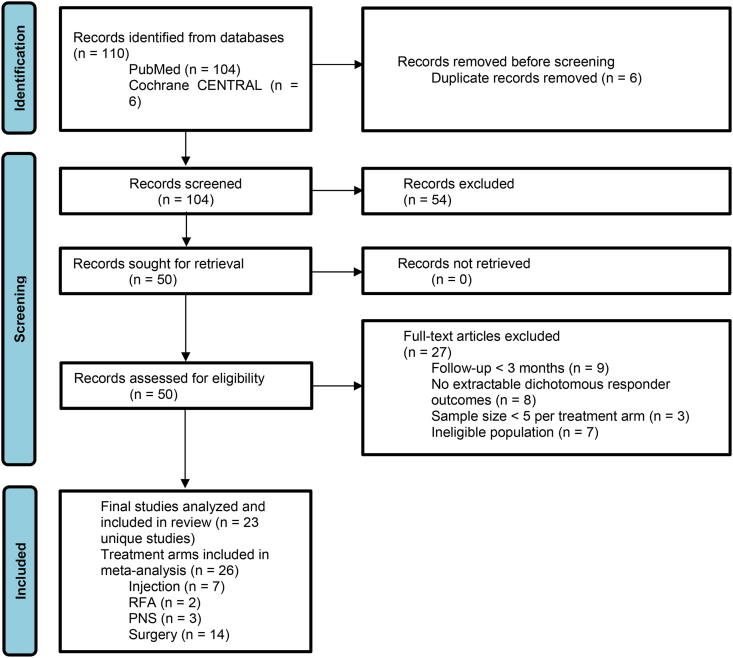
PRISMA flow diagram of study selection. Flowchart summarizing the identification, screening, eligibility assessment, and inclusion of studies evaluating interventions for cluneal nerve–related pain. Reasons for exclusion at the full-text stage are reported according to PRISMA guidelines (Page et al., 2021).

Titles and abstracts identified through the database search were screened independently by two reviewers (KC; JJ). Potentially eligible studies underwent full-text assessment for inclusion. Disagreements were resolved through discussion and consensus. The study selection process is summarized in a PRISMA flow diagram (Fig. 1).

### 2.5 Data extraction

Data were extracted at the treatment-arm level using a predefined data collection framework. The following variables were collected: study characteristics (author, year, study design), cluneal nerve involved (SCN, MCN, or mixed), treatment modality, number of patients per treatment arm, number of responders, follow-up duration, diagnostic criteria, and use of diagnostic nerve blocks.

Diagnostic work-up was recorded in a structured manner to capture how each study established the diagnosis and selected patients for treatment (e.g., clinical examination alone, diagnostic block, stimulation-based confirmation, or combinations thereof). Because outcome reporting was not uniform across the included studies, responder status was harmonized pragmatically. Whenever available, a responder was defined as a patient achieving at least 50% pain reduction. If this threshold was not reported, the dichotomous outcome judged by the original study authors to represent clinically meaningful improvement at the relevant follow-up time point was extracted. Accordingly, the pooled outcome represents a harmonized responder construct rather than a fully uniform endpoint.

### 2.6 Outcomes

The primary outcome was the pooled responder rate at ≥3 months follow-up. A predefined sensitivity analysis was performed including only studies with ≥6 months follow-up to evaluate durability of treatment effects. Subgroup analyses were conducted according to:

● treatment modality (surgery, injection therapy, radiofrequency ablation, peripheral nerve stimulation)
● surgical versus non-surgical treatment strategies
● nerve type (superior cluneal nerve vs middle cluneal nerve).

### 2.7 Risk of bias assessment

Methodological quality was assessed at the study level using the Newcastle-Ottawa Scale (NOS) for observational studies. Because data extraction and synthesis were performed at the treatment-arm level, risk-of-bias judgments were not assigned separately to individual treatment arms derived from the same study. Two reviewers (KC, JJ) evaluated study quality independently, with disagreements resolved by consensus. Because many included studies were uncontrolled case series or single-arm observational cohorts, the comparability domain was frequently not applicable or received no stars. Accordingly, NOS scores should be interpreted as a structured assessment of methodological reporting quality and selected risk-of-bias domains rather than as a definitive measure of internal validity across study designs. The completed risk-of-bias assessment will be provided in Supplementary Table S1.

### 2.8 Statistical analysis

All statistical analyses were performed in R (R Foundation for Statistical Computing, Vienna, Austria). Responder proportions were pooled using random-effects meta-analysis of proportions with logit transformation. Between-study variance was estimated using the restricted maximum likelihood (REML) method. A continuity correction of 0.5 was applied when necessary for studies with 0% or 100% event rates.

The primary analysis included treatment arms with follow-up of at least 3 months, representing the most consistently reported time threshold across studies. A predefined sensitivity analysis was restricted to studies reporting follow-up of at least 6 months to explore the durability of treatment response. Additional subgroup analyses were performed according to treatment modality, surgical versus non-surgical strategy, and cluneal nerve involved (SCN, MCN, or mixed cohorts).

Heterogeneity was quantified using the I^2^ statistic, and prediction intervals were calculated to estimate the expected range of effects in future clinical settings. Forest plots were used to visualize pooled responder rates.

Because several studies contributed more than one eligible treatment arm, the primary analysis was conducted at the treatment-arm level and therefore may not fully preserve statistical independence across all observations. To assess the robustness of the findings, a predefined sensitivity analysis was performed including only one treatment arm per study; in such cases, the arm with the largest sample size was retained. Subgroup analyses were considered exploratory and were not adjusted for multiple testing.

## 3 Results

### 3.1 Study characteristics

The database search yielded 110 records. After removal of duplicates, 104 studies remained for title and abstract screening. Following full-text review, 23 studies met the inclusion criteria and were included in the final analysis. The study selection process is illustrated in Fig. 1.

A total of 26 treatment arms including 628 patients with cluneal nerve-related pain were included in the meta-analysis. Most studies investigated superior cluneal nerve (SCN) entrapment, whereas a smaller number focused on middle cluneal nerve (MCN) neuropathy or mixed cohorts. Treatment modalities included surgical decompression (14 arms), local anesthetic injection therapy (7 arms), radiofrequency ablation (2 arms), and peripheral nerve stimulation (3 arms).

Most included studies were retrospective observational series, whereas a smaller number were prospective cohorts. Follow-up duration varied across studies, allowing pooled analyses at ≥3 months and ≥6 months. Study characteristics are summarized in Table 1. A formal study-level risk-of-bias assessment was performed and is presented in Supplementary Table S1.

**Table 1:** Characteristics of included treatment arms in the systematic review and meta-analysis.

ID	Year	Authors	Study Design	Nerve	Treatment	Responders/Total	Responder Definition	Follow-up (months)	Conclusion
	1) Group: Injection								
5	2014	Kuniya et al. (2014)	prospective	SCN	Local anesthetic block (procaine)	77/113	≥50% pain reduction	15.3	SCN-E is a common, often overlooked cause of LBP and leg symptoms. Most patients improve with nerve block injections.
7	2015	Kim et al. (2015)	retrospective	SCN	SCN block (not specified)	17/27	≥50% pain reduction	19.3	SCN-E can contribute to LBP in patients with vertebral compression fractures. Treatment with SCN blocks can significantly relieve pain.
13	2018	Yamauchi et al. (2018)	retrospective	SCN	Local anesthetic block (lidocaine)	8/12	≥50% pain reduction	26.4	Persistent or recurrent pain after lumbar disc surgery may be due to SCN-E, which can mimic lumbar spine pathology. Diagnosis and treatment with SCN blocks can improve symptoms.
14	2020	Sakamoto et al. (2020)	retrospective	Mixed	Local anesthetic block (lidocaine)	9/13	≥50% pain reduction	25.3	Paralumbar spine diseases such as SCN/MCN-E can coexist with lumbar disc herniation and contribute to LBP. Targeted nerve blocks can significantly reduce pain in affected patients.
15	2021	Fujihara et al. (2021a)	retrospective	MCN	Local anesthetic block (lidocaine)	41/50	≥50% pain reduction	18.4	MCN blocks can effectively relieve pain in many patients with MCN-E neuropathy and help confirm the diagnosis.
19	2022	Gautam et al. (2022)	prospective	SCN	Local anesthetic block (lidocaine)	20/25	≥50% pain reduction	6	A single SCN block results in significant pain relief in LBP patients with SCN-E for a period of 6 months.
22	2023	Wu et al. (2023)	prospective	SCN	Ultrasound-guided hydrodissection (5% dextrose)	28/36	Study-defined improvement	3	Ultrasound can accurately localize SCN branches, though nerve size is not useful for diagnosis. Most patients improve with ultrasound-guided dextrose hydrodissection, although recurrence may occur, especially in those with scoliosis.
	2) Group: RFA								
17	2021	Fujihara et al. (2021b)	retrospective	MCN	Radiofrequency (thermocoagulation (RFTC)	7/11	Study-defined improvement	6	RFTC may safely control intractable LBP due to MCN-E.
20	2022	Visnjevac et al. (2022)	retrospective	SCN	Radiofrequency ablation	36/46	≥50% pain reduction	3.2	Radiofrequency ablation of the superior cluneal nerve provided clinically meaningful pain reduction in most treated patients.
	3) Group: PNS								
24	2024	Lindley and Anders (2024)	retrospective	SCN	High-frequency neuromodulation	15/20	≥50% pain reduction	11	Neuromodulation targeting the SCN may reduce chronic LBP related to cluneal neuropathy.
26	2025	Mas et al. (2025)	retrospective	SCN	Peripheral nerve stimulation	4/6	≥50% pain reduction	3	Combined fluoroscopic and ultrasound guidance enables accurate placement of PNS leads and may improve pain outcomes in refractory cases.
25	2025	Skaribas et al. (2025)	retrospective	SCN	Peripheral nerve stimulation	20/21	≥50% pain reduction	6	Peripheral nerve stimulation can effectively treat SCN neuropathy causing focal pain that persists despite spinal cord stimulation. It provides substantial pain reduction and improved quality of life with minimal complications.
	4) Group: Surgery								
1	1997	Maigne and Doursounian (1997)	prospective	SCN	Surgical decompression	13/19	Study-defined improvement	38.4	SCN-E at the iliac crest is a treatable cause of chronic LBP and may respond to surgical release.
3	2013	Morimoto et al. (2013)	prospective	SCN	Surgical decompression	34/34	Study-defined improvement	10	Surgical decompression significantly improved pain in patients with confirmed SCN-E.
2	2013	Kim et al. (2013)	prospective	SCN	Surgical decompression	16/16	Study-defined improvement	12	Indocyanine green video angiography helps identify the SCN and confirm adequate decompression during surgery. This technique may improve the surgical treatment of SCN-E neuropathy.
4	2014	Kuniya et al. (2013)	prospective	SCN	Surgical decompression	12/19	≥50% pain reduction	15.3	Surgical decompression of the SCN can relieve symptoms in patients with persistent pain after nerve blocks. It is most effective in patients with shorter symptom duration and good response to diagnostic injections.
6	2015	(Kim et al., 2015)	retrospective	SCN	Surgical decompression	10/10	≥50% pain reduction	19.3	Surgical decompression of the SCN can significantly relieve LBP when nerve blocks provide only temporary relief. It is an effective treatment for SCN-E associated with vertebral compression fractures.
8	2016	Chiba et al. (2016)	retrospective	SCN	Surgical decompression	5/5	≥50% pain reduction	17.6	Surgical decompression of the SCN effectively relieved intermittent LBP caused by SCN-E when nerve blocks were only temporarily effective. SCN-E should be considered in patients whose LBP is triggered by standing or walking.
10	2017	Morimoto et al. (2017)	prospective	SCN	Surgical decompression	52/52	Study-defined improvement	41.3	Surgical decompression of the SCN provides good long-term relief for SCN-E neuropathy. Adequate decompression of multiple nerve branches during the first surgery may reduce recurrence.
11	2017	Iwamoto et al. (2017)	prospective	SCN	Surgical decompression	8/8	≥50% pain reduction	28	Surgical decompression of the SCN can significantly relieve persistent LBP after lumbar fusion surgery. SCN-E should be considered as a potential cause of failed back surgery syndrome.
9	2017	Kokubo et al. (2017)	prospective	SCN	Surgical decompression	7/7	≥50% pain reduction	21.4	Surgical decompression of the SCN under local anesthesia can effectively relieve LBP and buttock pain in very elderly patients with SCN-E. Minimally invasive peripheral nerve surgery is a useful treatment option when conservative therapy fails.
12	2018	Matsumoto et al. (2018)	prospective	MCN	Surgical decompression	11/11	≥50% pain reduction	10.5	Surgical decompression (MCN neurolysis) under local anesthesia effectively relieves LBP caused by MCN-E. MCN-E should be considered in patients with unexplained or refractory LBP.
16	2021	Fujihara et al. (2021a)	retrospective	MCN	Surgical decompression	9/9	≥50% pain reduction	18.4	Surgical decompression is an effective treatment for patients with MCN-E whose symptoms do not respond to conservative therapy.
18	2022	Kim et al. (2022a)	retrospective	Mixed	Peripheral nerve surgery outcomes	22/22	≥50% pain reduction	6	Surgical decompression for CN-E significantly improves pain and function. Overall patient satisfaction with the surgical outcome is high.
21	2023	Tajiri et al. (2023)	retrospective	MCN	Surgical decompression	30/30	≥50% pain reduction	40.4	Surgical release of the MCN provides effective long-term relief of LBP caused by MCN-E. However, recurrence may occur, and additional surgery can restore symptom improvement.
23	2024	Kim et al. (2024)	prospective	MCN	Surgical decompression	6/6	≥50% pain reduction	11.7	Surgical treatment (MCN neurectomy) can effectively relieve LBP caused by MCN-E. Pathological findings support nerve compression as the underlying cause of symptoms.

The table summarizes study design, treatment modality, cluneal nerve involved, responder definition, follow-up duration, and diagnostic block use for all included treatment arms.

SCN = superior cluneal nerve; MCN = middle cluneal nerve; RFA = radiofrequency ablation; PNS = peripheral nerve stimulati

### 3.2 Overall pooled responder rates

**Fig. 2:**
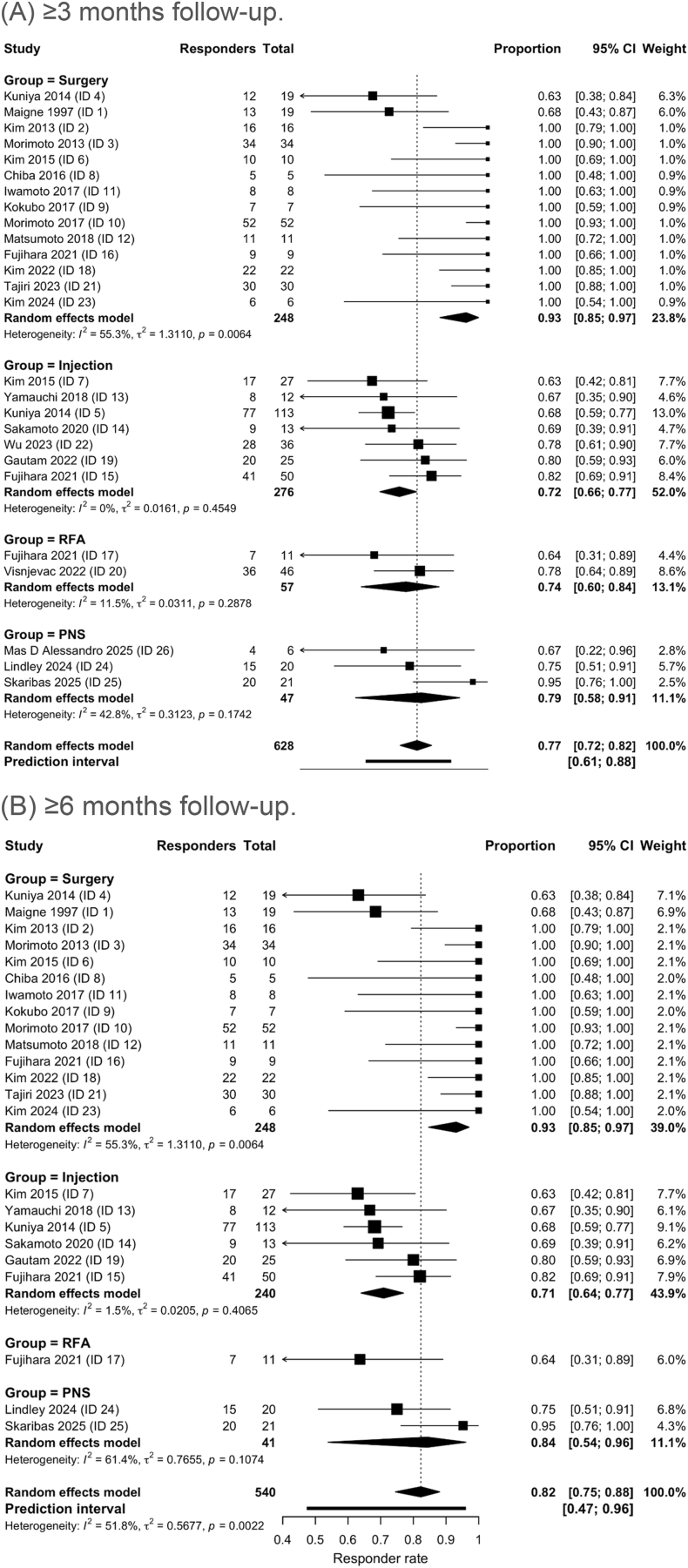
Forest plots of pooled responder rates stratified by treatment modality. (A) ≥3 months follow-up. Forest plot of pooled responder rates stratified by treatment modality at ≥3 months of follow-up. Individual treatment arms are displayed as weighted squares with 95% confidence intervals. Pooled estimates are represented by diamonds derived from random-effects meta-analysis. Interventions include surgical decompression, injection therapy, radiofrequency ablation (RFA), and peripheral nerve stimulation (PNS). (B) ≥6 months follow-up. Fig. 2B. Forest plot of pooled responder rates stratified by treatment modality at ≥6 months of follow-up. Squares represent individual treatment arms weighted by inverse variance, with horizontal lines indicating 95% confidence intervals. Diamonds represent pooled responder rates calculated using a random-effects model. Subgroups include surgery, injection therapy, RFA, and PNS.

Across all treatment arms, 517 of 628 patients were classified as responders. Random-effects meta-analysis demonstrated a pooled responder rate of 78.3% (95% CI 72.9-82.9) at ≥3 months of follow-up. In the sensitivity analysis including studies reporting ≥6 months follow-up, 449 of 540 patients were responders, corresponding to a pooled responder rate of 82.9% (95% CI 75.3-88.6) (Fig. 2B). The slightly higher pooled responder rate at ≥6 months most likely reflects differences in the subset of studies contributing longer-term data rather than a true improvement over time. Importantly, modality-specific conclusions regarding durability are limited, as follow-up reporting was inconsistent across treatment categories and only a subset of studies contributed ≥6-month data. In leave-one-out analyses, pooled responder rates remained within a narrow range at both ≥3 months and ≥6 months, supporting the robustness of the findings. The prediction interval ranged from 61.0% to 88.2% for the ≥3-month analysis and from 20.5% to 99.9% for the ≥6-month analysis, indicating substantially greater uncertainty in the longer-term subset. Sensitivity analyses including only one treatment arm per study yielded very similar pooled responder rates (≥3 months: 77.4%, 95% CI 71.9-82.0; ≥6 months: 82.4%, 95% CI 74.2-88.4), indicating that the inclusion of multiple arms from individual studies did not materially influence the results.

### 3.3 Responder rates by treatment modality

When stratified by treatment modality, surgical decompression demonstrated the highest pooled responder rate, reaching 93.1% (95% CI 84.9-97.0). Injection therapy showed a pooled responder rate of 72.4% (95% CI 65.9-78.1). Radiofrequency ablation demonstrated a pooled responder rate of 75.1% (95% CI 62.1-84.7). Peripheral nerve stimulation showed a pooled responder rate of 80.1% (95% CI 58.5-92.0). Forest plots illustrating pooled responder rates stratified by treatment modality at ≥3 months and ≥6 months are shown in Fig. 2A and B.

When grouped by treatment strategy, surgical treatment was associated with higher pooled responder proportions than non-surgical interventions. At ≥3 months of follow-up, 235 of 248 surgically treated patients were classified as responders compared with 282 of 380 patients treated with non-surgical interventions. The pooled responder rate was 93.1% (95% CI 84.9-97.0) for surgical treatment and approximately 74-75% for non-surgical interventions. However, these groups were not directly comparable, as treatment allocation was non-randomized and likely influenced by diagnostic certainty, prior treatment failure, and patient selection.

Similar differences were observed at ≥6 months, where 235 of 248 patients in the surgical group and 214 of 292 patients in the non-surgical group were classified as responders.

### 3.4 Responder rates by cluneal nerve

Subgroup analysis demonstrated high responder rates for both SCN and MCN interventions (Table 2). SCN-targeted treatments showed consistently high response rates across studies. MCN cohorts also demonstrated favorable outcomes, although the number of available studies was smaller. Mixed cohorts including both SCN and MCN pathology showed comparable pooled outcomes.

**Table 2:** Pooled responder rates overall and stratified by cluneal nerve and treatment modality.

Group	Follow-up	Arms, n	Responders/Total	Pooled responder rate, % (95%% CI)	I (Wu et al., 2023), %
Overall	≥3 months	26	517/628	78.3 (72.9-82.9)	28.7
Overall	≥6 months	23	449/540	82.9 (75.3-88.6)	59.9
SCN	≥3 months	18	382/476	75.6 (69.8-80.6)	17.6
SCN	≥6 months	15	314/388	81.3 (71.3-88.4)	61.9
MCN	≥3 months	6	104/117	87.8 (71.1-95.4)	50.3
MCN	≥6 months	6	104/117	87.8 (71.1-95.4)	50.3
Surgery	≥3 months	14	235/248	93.1 (84.9-97.0)	52.9
Surgery	≥6 months	14	235/248	93.1 (84.9-97.0)	52.9
Injection	≥3 months	7	200/276	72.4 (65.9-78.1)	14.3
Injection	≥6 months	6	172/240	71.6 (64.3-77.9)	16.4
RFA	≥3 months	2	43/57	75.1 (62.1-84.7)	0.0
RFA	≥6 months	1	7/11	63.6 (33.9-85.7)	0.0
PNS	≥3 months	3	39/47	80.1 (58.5-92.0)	32.1
PNS	≥6 months	2	35/41	86.3 (50.7-97.5)	63.4

Responder rates were analyzed at ≥3 and ≥ 6 months of follow-up. Pooled proportions were estimated using random-effects meta-analysis with logit transformation and restricted maximum likelihood estimation of between-study variance. SCN = superior cluneal nerve; MCN = middle cluneal nerve; RFA = radiofrequency ablation; PNS = peripheral nerve stimulation. I^2^ represents the proportion of total variability attributable to between-study heterogeneity.

## 4 Discussion

### 4.1 Principal findings

This systematic review and meta-analysis found that targeted treatment of cluneal nerve–related pain was associated with favorable pooled responder rates across all included modalities. The pooled responder rate was 78.3% (95% CI 72.9–82.9) at ≥3 months and 82.9% (95% CI 75.3–88.6) at ≥6 months of follow-up. Surgical decompression was associated with the highest pooled responder proportion, whereas injection therapy, radiofrequency ablation, and peripheral nerve stimulation were also associated with clinically meaningful improvement in selected patients. However, these findings should not be interpreted as proof of comparative superiority, because the available evidence is predominantly observational, non-randomized, and subject to heterogeneity in diagnostic work-up, outcome definition, and follow-up duration. The slightly higher pooled responder rate at ≥6 months most likely reflects differences in the subset of studies contributing longer-term data rather than true improvement over time. The wide prediction intervals, particularly in the ≥6-month analysis, highlight the variability of treatment effects across clinical settings and underscore the uncertainty of long-term outcomes.

Non-interventional conservative approaches, such as physiotherapy, stretching, mobilization, and soft tissue techniques, may play a role in the management of cluneal nerve-related pain in clinical practice. However, these strategies were not included in the present quantitative synthesis because they were inconsistently reported, not standardized across studies, and rarely presented as extractable treatment arms with clearly defined responder outcomes. Accordingly, no pooled conclusions regarding their effectiveness can be drawn from the available data.

### 4.2 Interpretation of findings

From a clinical perspective, these findings support a stepwise treatment framework: (1) careful clinical identification of cluneal nerve–mediated pain, (2) confirmatory diagnostic blocks as used in study protocols, (3) less invasive treatments (injections, RFA, or PNS) in appropriately selected patients, and (4) surgical decompression/neurolysis for persistent or recurrent symptoms. However, the pooled estimates should primarily be interpreted as evidence that appropriately selected patients frequently improve after targeted treatment, rather than as proof of superiority of one modality over another. Importantly, pooled responder proportions across modalities reflect fundamentally different patient populations, diagnostic thresholds, and treatment-selection pathways. Patients undergoing surgery were often selected after failure of conservative or interventional treatment and frequently had stronger clinical or block-based evidence supporting a cluneal nerve pain generator. In this sense, surgical cohorts are typically enriched for patients with a higher likelihood of true entrapment neuropathy at baseline. Therefore, comparisons between modalities should be interpreted cautiously, as the underlying populations are not directly comparable. (Isu et al., 2018; Karl et al., 2022; Anderson et al., 2022; Gill et al., 2022) In addition, the different treatment modalities address distinct aspects of the condition. Surgical decompression targets the presumed anatomical entrapment, whereas injection therapy, radiofrequency procedures, and neuromodulation primarily modulate nociceptive signaling without necessarily resolving the structural constraint. These differences in mechanism, patient selection, and diagnostic certainty are central when interpreting pooled responder rates. Cluneal neuropathy remains under-recognized and is frequently misattributed to degenerative spinal pathology, which may lead to persistent symptoms even after spine-directed interventions. This is particularly evident in cohorts following lumbar surgery (Yamauchi et al., 2018) or fusion, (Iwamoto et al., 2017) as well as in older patients with multimorbidity. (Fujihara et al., 2024) Our study contributes by moving beyond narrative syntheses (Isu et al., 2018; Karl et al., 2022; Anderson et al., 2022; Gill et al., 2022) and providing a quantitative pooled estimate of responder rates by modality and follow-up threshold, based on structured extraction of diagnostic criteria, follow-up duration, responder definitions, complications, and reinterventions. Because cluneal neuropathy frequently coexists with other spinal or peri-spinal pain generators (Fujihara et al., 2024; Kim et al., 2015; Sakamoto et al., 2020; Fujihara et al., 2021a), pooled treatment effects likely reflect a combination of true cluneal-targeted benefit and variable residual confounding by co-pathology. These studies support the central premise of the present meta-analysis: when cluneal neuropathy is correctly identified and treated with an appropriate modality, clinically meaningful improvement is common.

### 4.3 Clinical criteria and provocative signs

Multiple studies emphasize that diagnosis is primarily clinical, typically characterized by focal tenderness/trigger points along the posterior iliac crest (often ∼7 cm from the midline for SCN) with reproduction of the patient's typical pain and radiating symptoms. (Isu et al., 2018) (Kuniya et al., 2014) (Iwamoto et al., 2017) (Miki et al., 2019) Miki et al. contributed important clinical phenotyping by describing characteristic features of LBP attributable to SCN entrapment, which is relevant for improving pre-test probability before blocks or procedures. (Miki et al., 2019) Kuniya et al.’s prospective work is particularly important for the field because it operationalized diagnostic criteria and examined prevalence and symptom patterns in a large LBP cohort, establishing cluneal nerve disorder as a clinically detectable entity rather than a purely “diagnosis of exclusion”. (Kuniya et al., 2014)

### 4.4 Diagnostic blocks and their implications

Several interventional and surgical pathways use diagnostic blocks to confirm the pain generator before escalation. This has two implications for our pooled outcomes: 1) response rates may be inflated because block-positive patients represent an enriched subgroup, and 2) studies not requiring block confirmation may include more diagnostic heterogeneity, explicitly discussed in major narrative/scoping reviews. (Karl et al., 2022; Anderson et al., 2022; Gill et al., 2022) Importantly, Takada et al. showed that painDETECT did not reliably classify cluneal nerve - mediated LBP as neuropathic, implying that questionnaire-based triage cannot replace targeted examination and confirmatory maneuvers. (Takada et al., 2024), supporting the diagnostic hierarchy reflected across the clinical literature consisting of clinical localization and an optional but often used confirmatory block. In patients with failed lumbar disc surgery, undiagnosed peripheral nerve disorders including cluneal nerve-related pain generators may contribute to persistent postoperative symptoms, and targeted diagnostic blocks followed by nerve-focused treatments can yield meaningful improvement in selected patients. (Yamauchi et al., 2018)

### 4.5 Ultrasound and imaging-based confirmation

Ultrasound has become a key tool for diagnosing and treating SCN pathology. Multiple studies show that high-resolution ultrasound enables accurate visualization of the nerve and supports image-guided interventions such as diagnostic blocks and hydrodissection. Overall, ultrasound guidance improves targeting accuracy and may reduce false-negative blocks, helping explain the consistency of outcomes seen in non-surgical treatments. (Wu et al., 2023; Bodner et al., 2016; Wu et al., 2022; Luo and Zhao, 2024).

### 4.6 Modality-specific discussion

1) Injection therapy and hydrodissection (including dextrose)

Perineural injection can function as 1) a diagnostic test (local anesthetic block), 2) a short-term therapeutic intervention (anesthetic ± steroid), or 3) a mechanical/biological intervention when delivered as hydrodissection (separating nerve from adhesions/fascial planes) or when using dextrose solutions proposed to modulate neurogenic inflammation. Injection-based interventions offer several practical advantages, as they are minimally invasive, repeatable, and generally well tolerated, making them suitable for older patients and those with relevant comorbidities. In addition to their therapeutic role, perineural injections can serve an important diagnostic function by helping to confirm the cluneal nerve as the primary pain generator, thereby supporting subsequent clinical decision-making. The relevance of cluneal nerve pathology also extends to LDH populations with coexisting paralumbar spine diseases. In such cases, treatment directed solely at disc pathology may fail to address concomitant peripheral pain generators. Sakamoto et al. demonstrated that targeted block therapy for paralumbar spine disease entities, including cluneal nerve involvement, can improve outcomes in LDH patients, underscoring the importance of diagnostic precision and identification of coexisting pain sources. (Sakamoto et al., 2020) However, the durability of analgesic effects after injection therapy is frequently limited, and many studies report relatively short follow-up intervals, with relapse and reintervention rates inconsistently documented. (Bodner et al., 2016; Luo and Zhao, 2024; Kiral et al., 2024) Moreover, diagnostic enrichment through the inclusion of block-responsive patients may lead to overestimation of apparent treatment effectiveness and complicates meaningful comparisons between injection-based approaches and other therapeutic modalities. (Karl et al., 2022; Gill et al., 2022) Interpretation of pooled effects is further constrained by substantial heterogeneity in injectates and techniques, ranging from local anesthetic alone to corticosteroid mixtures and dextrose-based hydrodissection (Yildirim, 2024), which precludes robust mechanistic conclusions and limits comparability across studies. Our pooled non-surgical responder estimates are consistent with injection therapy being a meaningful option for many patients, but the injection literature's variability in definitions and follow-up likely contributes to between-study heterogeneity and limits durability conclusions.

2) Radiofrequency procedures (RFA/related techniques)

By interrupting nociceptive transmission radiofrequency-based interventions offer the potential for more durable analgesia than injection therapies while remaining minimally invasive and feasible as outpatient procedures, making them an appealing intermediate option before considering surgical decompression when diagnostic confidence is adequate. They may be also particularly valuable in clinical scenarios where repeated perineural injections provide only transient or diminishing benefit. Visnjevac et al. reported a large series of patients treated with SCN RFA and described the technique as a minimally invasive approach informed by anatomy/neurosurgical understanding. (Visnjevac et al., 2022) At the same time, the current evidence base for radiofrequency treatment of cluneal nerve entrapment is largely observational, with substantial variability in patient selection criteria and procedural techniques across studies. Follow-up durations are often limited, and relapse or reintervention rates are inconsistently reported, which is clinically relevant given the potential for recurrence of nerve-mediated pain over time. Moreover, effective and safe radiofrequency treatment depends on precise anatomical targeting of the cluneal nerve, and variability in localization and confirmation strategies - such as the use of sensory stimulation versus purely anatomical placement - may contribute to heterogeneity in outcomes and complicate cross-study comparisons. (Wu et al., 2022; Visnjevac et al., 2022) The pooled RFA response in our synthesis aligns with RFA being an effective option in selected patients, but the evidence base remains sensitive to technique and diagnostic enrichment. Consequently, pooled estimates should not be interpreted as stable “true effects” across settings until more standardized prospective cohorts exist. (Karl et al., 2022; Gill et al., 2022).

3) Peripheral nerve stimulation (PNS) and neuromodulation strategies

PNS aims to modulate afferent signaling and central pain processing without permanently destroying nerve tissue. It can be positioned as reversible escalation for refractory cases or for patients who wish to avoid surgery. Alessandro et al. reported a clinical case series using a combined fluoroscopy and ultrasound technique for peripheral SCN stimulation, emphasizing practical implantation workflow for intractable LBP attributed to SCN involvement. (Mas et al., 2025) Skaribas et al. reported a retrospective series of PNS for SCN neuropathy used as an adjunct when patients with successful spinal cord stimulation (SCS) developed focal peripheral pain not adequately addressed by reprogramming, demonstrating a role for PNS within complex neuromodulation pathways. (Skaribas et al., 2025) Lindley et al. contribute to the device-based neuromodulation landscape with an implanted neurostimulator approach at the SCN powered via high-frequency electromagnetic coupling, illustrating a more technology-driven, frequently overlooked, direction for sustained therapy. (Lindley and Anders, 2024) Peripheral nerve stimulation offers several conceptual advantages, as it is reversible and adjustable and may therefore be particularly attractive for patients with elevated surgical risk or those who prefer a non-destructive, titratable therapy. It is especially relevant for focal cluneal nerve-mediated pain that persists despite broader neuromodulation strategies such as spinal cord stimulation, where peripheral targeting can complement central neuromodulation to address residual localized symptoms. (Skaribas et al., 2025) Nevertheless, the current evidence supporting peripheral nerve stimulation for cluneal neuropathy remains at an early stage and is largely derived from small case series and retrospective designs, rendering reported effect sizes vulnerable to publication bias and selection bias. Device-related considerations, including lead migration, infection risk, explantation, and hardware-related constraints such as battery or receiver management, require systematic and standardized reporting, which has been inconsistent across early reports. In addition, heterogeneity in stimulation paradigms, trial protocols, and anatomical targeting complicates meaningful pooling of outcomes and limits comparability between studies. (Abd-Elsayed and Gyorfi, 2024; Skaribas et al., 2025) Our pooled PNS responder estimate supports neuromodulation as a credible option within the therapeutic spectrum, but the small-study nature of PNS evidence means that pooled estimates must be interpreted as preliminary and hypothesis-generating. The modality may be best positioned as an escalation option where diagnostic confidence is high and non-device interventions have not provided durable relief.

4) Surgery: decompression/neurolysis and the role of “definitive” anatomical treatment

Surgical decompression or neurolysis directly targets the presumed anatomical entrapment mechanism of the cluneal nerves and may therefore offer durable symptomatic benefit in appropriately selected patients. This approach is particularly relevant in clinical scenarios characterized by missed or delayed diagnosis, such as persistent or recurrent low back pain following spine surgery, where undiagnosed cluneal nerve entrapment has been identified as a treatable contributor to ongoing symptoms. (Yamauchi et al., 2018; Iwamoto et al., 2017) Similarly, in post-lumbar fusion pain, SCN-E can contribute to residual LBP, and targeted treatment has been associated with symptom improvement, emphasizing that cluneal neuropathy represents a treatable diagnosis even in complex post-surgical pain states. (Iwamoto et al., 2017) In clinically complex settings, Yamauchi et al. showed that undiagnosed peripheral nerve disorders including cluneal nerve pathology can be relevant in failed lumbar disc surgery patients and that nerve-directed approaches may improve symptoms in selected individuals. (Yamauchi et al., 2018) Iwamoto et al. similarly reported that identifying and treating SCN entrapment improved LBP in patients after lumbar fusion surgery, reinforcing the principle that surgery can address a peripheral generator when spine-directed interventions do not resolve symptoms. (Iwamoto et al., 2017) Surgical relevance is further supported in older adults and fracture-related pain phenotypes. Kim et al. reported outcomes in patients with vertebral compression fractures complicated by SCN entrapment neuropathy, emphasizing that fracture-associated biomechanical changes may coexist with peripheral nerve entrapment and that targeted therapy can improve pain/disability metrics. (Kim et al., 2015) Fujihara et al. extended this concept by emphasizing PLSDs - including cluneal nerve entrapment - as treatable causes of LBP in older adults, a population in which surgical decisions require careful balancing of invasiveness and benefit. (Fujihara et al., 2024; Fujihara et al., 2021a) In specialized centers, perioperative safety profiles for peripheral nerve and paralumbar spine disease procedures have been reported as acceptable, although the heterogeneity of reporting frameworks limits precise estimation of procedure-specific risks. (Kim et al., 2022b) Conversely, surgical intervention is inherently more invasive and entails perioperative risk, which is of particular relevance in frail or very old patient populations who frequently present with multiple comorbidities. Surgical outcomes are highly dependent on diagnostic certainty, undertaking decompression in diagnostically heterogeneous cohorts risks addressing an incorrect or only partially contributory pain generator, thereby diminishing apparent effectiveness. Furthermore, complication reporting specific to cluneal nerve surgery is not uniformly standardized (e.g., Clavien–Dindo thresholds), and while broader paralumbar spine disease complication datasets provide useful contextual safety information, they are not fully procedure-specific and therefore limit granular risk stratification for cluneal nerve-targeted operations. (Kim et al., 2022a; Kim et al., 2022b) The very high pooled surgical responder rates in our synthesis likely reflect both a genuine treatment effect and diagnostic enrichment (block-positive or clinically classic cases, often after failed conservative measures). Accordingly, the results support surgery as a highly effective option for appropriately selected patients, while reinforcing the need for standardized prospective comparative data to address confounding by indication.

### 4.7 Limitations

Several limitations should be considered when interpreting the findings of this meta-analysis. First, the available evidence is predominantly based on retrospective case series and observational cohort studies, with no randomized controlled trials identified. As a result, pooled responder rates should not be interpreted as causal estimates of comparative treatment effectiveness.

Second, confounding by indication is highly likely. Patients undergoing surgical treatment were often selected after more stringent diagnostic confirmation and/or failure of prior conservative or interventional therapies, and therefore may represent a subgroup with a higher baseline likelihood of true cluneal nerve entrapment. Accordingly, comparisons between surgical and non-surgical treatments are inherently limited and should be interpreted cautiously.

Third, diagnostic criteria varied substantially across studies. Some studies relied primarily on clinical examination findings, whereas others incorporated diagnostic nerve blocks, stimulation-based confirmation, or combinations thereof. This heterogeneity limits comparability across study populations and introduces uncertainty regarding whether pooled estimates reflect a uniform clinical entity.

Fourth, responder definitions were not fully standardized. While many studies used a ≥50% reduction in pain intensity, others relied on study-specific definitions of clinical improvement. Therefore, the pooled outcome represents a pragmatically harmonized responder construct rather than a strictly uniform endpoint.

Fifth, follow-up duration was inconsistent, and longer-term data were available only for a subset of studies. Consequently, conclusions regarding durability of treatment effects - particularly at the level of individual modalities - remain limited.

Sixth, data were analyzed at the treatment-arm level. Because some studies contributed multiple treatment arms, statistical independence between all observations cannot be fully guaranteed, although sensitivity analyses suggested that this did not materially affect the results.

Finally, adverse event reporting was inconsistent and insufficient for quantitative synthesis, and the possibility of publication bias and small-study effects - particularly in smaller cohorts and emerging modalities such as PNS - cannot be excluded.

## 5 Conclusion

Targeted treatment of superior and middle cluneal nerve entrapment is associated with high pooled responder rates, with favorable outcomes observed at both ≥3 and ≥ 6 months of follow-up. Surgical decompression demonstrated the highest pooled responder proportion, whereas minimally invasive interventional treatments also showed clinically meaningful benefit in selected patients.

However, these findings should not be interpreted as definitive evidence of superiority of one treatment modality over another, given the predominantly observational, non-comparative nature of the available evidence, heterogeneity in diagnostic criteria and outcome definitions, and differences in patient selection across studies. Future prospective studies using standardized diagnostic pathways, uniform outcome definitions, and comparative designs are needed to better define the role of each treatment strategy.

## Declaration of competing interest

The authors declare that they have no known competing financial interests or personal relationships that could have appeared to influence the work reported in this paper.

## List of Abbreviations

LDH: Lumbar Disc Herniation
LBP: Low Back Pain
MCN: Middle Cluneal Nerve
MCN-E: Middle Cluneal Nerve Entrapment
PLSD: Paralumbar Spine Diseases
PNS: Peripheral Nerve Stimulation
RFA: Radiofrequency Ablation
SCN: Superior Cluneal Nerve
SCN-E: Superior Cluneal Nerve Entrapment
