# Supplementary material for: Treatment outcomes for cluneal neuropathy: a systematic review and meta-analysis
Source: Brain Spine. 2026 Apr 27;6:106062. doi: 10.1016/j.bas.2026.106062 (PMC13195346; doi:10.1016/j.bas.2026.106062)
Supplement: Multimedia component 1 [file mmc1.docx]

## S1: Risk of bias assessment

|  |  |  |  |  | Selection |  |  |  | Comparability |  |  | Outcome |  |  |
| --- | --- | --- | --- | --- | --- | --- | --- | --- | --- | --- | --- | --- | --- | --- |
| ID | Author | Year | Modality | Nerve | Represent ativeness | Comparator | Ascertainment of exposure | Outcome not present  at start | Control for confounders (max 2 stars) | Assessment of outcome | | Follow-up long enough | Adequacy of follow-up | Total  Stars (0–9) |
| 1 | Maigne | 1997 | Surgery | SCN | 0 | 0 | 1 | 1 | 0 | 1 | | 1 | 0 | 4 |
| 2 | Kim | 2013 | Surgery | SCN | 1 | 0 | 1 | 1 | 0 | 1 | | 1 | 1 | 6 |
| 3 | Morimoto | 2013 | Surgery | SCN | 1 | 0 | 1 | 1 | 0 | 1 | | 1 | 1 | 6 |
| 4 | Kuniya | 2014 | Injection/S urgery | SCN | 1 | 1 | 1 | 1 | 1 | 1 | | 1 | 1 | 8 |
| 5 | Kim | 2015 | Injection/  Surgery | SCN | 1 | 0 | 1 | 1 | 0 | 1 | | 1 | 1 | 6 |
| 6 | Chiba | 2016 | Surgery | SCN | 0 | 0 | 1 | 1 | 0 | 1 | | 1 | 1 | 5 |
| 7 | Kokubo | 2017 | Surgery | SCN | 1 | 0 | 1 | 1 | 1 | 1 | | 1 | 0 | 6 |
| 8 | Morimoto | 2017 | Surgery | SCN | 1 | 0 | 1 | 1 | 0 | 1 | | 1 | 1 | 6 |
| 9 | Iwamoto | 2017 | Surgery | SCN | 0 | 0 | 1 | 1 | 0 | 1 | | 1 | 1 | 5 |
| 10 | Matsumoto | 2018 | Surgery | MCN | 0 | 0 | 1 | 1 | 0 | 1 | | 1 | 1 | 5 |
| 11 | Yamauchi | 2018 | Injection | SCN | 0 | 0 | 1 | 1 | 0 | 1 | | 1 | 1 | 5 |
| 12 | Sakamoto | 2020 | Injection | Mixed | 1 | 0 | 1 | 1 | 0 | 1 | | 0 | 1 | 5 |
| 13 | Fujihara | 2021 | Injection/  Surgery | MCN | 1 | 0 | 1 | 1 | 0 | 1 | | 1 | 0 | 5 |
| 14 | Fujihara | 2021 | RFA | MCN | 0 | 0 | 1 | 1 | 0 | 1 | | 1 | 1 | 5 |
| 15 | Kim | 2022 | Surgery | Mixed | 0 | 0 | 1 | 1 | 0 | 1 | | 1 | 0 | 4 |
| 16 | Gautam | 2022 | Injection | SCN | 1 | 0 | 1 | 1 | 0 | 1 | | 1 | 1 | 6 |
| 17 | Visnjevac | 2022 | RFA | SCN | 1 | 0 | 1 | 0 | 0 | 1 | | 1 | 0 | 4 |
| 18 | Tajiri | 2023 | Surgery | MCN | 1 | 0 | 1 | 1 | 0 | 1 | | 1 | 1 | 6 |
| 19 | Wu | 2023 | Injection | SCN | 1 | 0 | 1 | 1 | 0 | 1 | | 0 | 1 | 5 |
| 20 | Kim | 2024 | Surgery | MCN | 1 | 0 | 1 | 1 | 0 | 1 | | 1 | 1 | 6 |
| 21 | Lindley | 2024 | PNS | SCN | 1 | 0 | 1 | 1 | 0 | 1 | | 1 | 1 | 6 |
| 22 | Skaribas | 2025 | PNS | SCN | 1 | 0 | 1 | 1 | 0 | 1 | | 1 | 1 | 6 |
| 23 | Mas D  Alessandro | 2025 | PNS | SCN | 0 | 0 | 1 | 1 | 0 | 1 | | 1 | 0 | 4 |

**Table 3. Newcastle–Ottawa Scale (NOS) quality assessment of included studies.** The methodological quality of the included observational studies was evaluated using the Newcastle–Ottawa Scale (NOS). This tool assesses risk of bias across three domains: selection of the study population (maximum 4 stars), comparability of study groups (maximum 2 stars), and outcome assessment (maximum 3 stars), with a total possible score of 0–9 stars. Higher scores indicate better methodological quality and lower risk of bias. For each study, stars were assigned according to the representativeness of the cohort, presence of a comparator group, ascertainment of the intervention, confirmation that the outcome was not present at baseline, control for confounders, method of outcome assessment, adequacy of follow-up duration, and completeness of follow-up. Because most included studies were single-arm case series or observational cohorts without comparator groups, the comparability domain generally received no stars, resulting in most studies achieving 4–6 stars overall. *Abbreviations*: **SCN** = superior cluneal nerve; **MCN** = middle cluneal nerve; **RFA** = radiofrequency ablation; **PNS** = peripheral nerve stimulation.

## S2: Full Search Strategy

(

"cluneal nerve"[Title/Abstract] OR "cluneal nerves"[Title/Abstract] OR

"superior cluneal nerve"[Title/Abstract] OR "superior cluneal nerves"[Title/Abstract] OR

"middle cluneal nerve"[Title/Abstract] OR "middle cluneal nerves"[Title/Abstract] OR

"medial superior cluneal nerve"[Title/Abstract] OR

"SCN"[Title/Abstract] OR "MCN"[Title/Abstract]

)

AND

(

entrap*[Title/Abstract] OR neuropath*[Title/Abstract] OR neuralgia[Title/Abstract] OR "nerve entrapment"[Title/Abstract]

)

AND

(

surg*[Title/Abstract] OR decompression[Title/Abstract] OR neurolysis[Title/Abstract] OR

"radiofrequency"[Title/Abstract] OR ablation[Title/Abstract] OR thermocoagulation[Title/Abstract] OR

stimulation[Title/Abstract] OR "peripheral nerve stimulation"[Title/Abstract] OR PNS[Title/Abstract] OR injection*[Title/Abstract] OR block*[Title/Abstract]
